# Salivary gland ultrasonography as a predictor of clinical activity in Sjögren’s syndrome

**DOI:** 10.1371/journal.pone.0182287

**Published:** 2017-08-04

**Authors:** Tania Fidelix, Adriano Czapkowski, Sergio Azjen, Adagmar Andriolo, Virginia F. M. Trevisani

**Affiliations:** 1 Department of Evidence-Based Medicine, Universidade Federal de São Paulo (UNIFESP), São Paulo, SP, Brazil; 2 Department of Radiology, UNIFESP, São Paulo, SP, Brazil; 3 Department of Laboratory Medicine, UNIFESP, São Paulo, SP, Brazil; University College London, UNITED KINGDOM

## Abstract

**Purpose:**

Primary Sjögren’s syndrome is a multisystem autoimmune disease characterized by hypofunction of salivary and lacrimal glands and possible multi-organ system manifestations. Over the past 15 years, three sets of diagnostic criteria have been proposed, but none has included salivary gland ultrasonography. However, recent studies support its role in the diagnosis and prognostic evaluation of patients with Sjögren’s syndrome. This study aimed to determine the value of salivary gland ultrasonography in the diagnosis and prognosis of Sjögren’s syndrome by relating ultrasonography severity scores to clinical and laboratory data.

**Methods:**

Seventy patients who fulfilled the 2002 American-European Consensus Group diagnostic criteria for primary Sjögren’s syndrome were selected from 84 patients receiving care in specialized outpatient clinics at our institution from November 2013 to May 2016. Their serology, European League Against Rheumatism Sjögren’s syndrome disease activity index (ESSDAI), salivary flow rate, immunoglobulin G, and salivary and serum beta-2 microglobulin levels were measured. Salivary gland ultrasonography was performed by an experienced radiologist, using scores of 1–4 to classify salivary gland impairment.

**Results:**

Salivary gland ultrasonography scores of 1 or 2 were associated with an ESSDAI < 5. Ultrasonography scores of 3 or 4 were associated with an ESSDAI ≥5 (p = 0.064), a positive antinuclear antibody test (p = 0.006), positive anti-Ro/SSA antibodies (p = 0.003), positive anti-La/SSB antibodies (p = 0.077), positive rheumatoid factor (p = 0.034), and immunoglobulin G levels > 1600 mg/dL (p = 0.077). Salivary flow rate was lower in patients with scores 3 or 4 (p = 0.001).

**Conclusion:**

This study provides further evidence that salivary gland ultrasonography can be used not only for diagnosis but also for prognostic evaluation of primary Sjögren’s syndrome. These findings confirm what has been reported in the literature. However, further analyses involving larger matched samples are required to support this finding and include salivary gland ultrasonography as part of the diagnostic criteria for Sjögren’s syndrome.

## Introduction

Primary Sjögren’s syndrome (pSS) is a systemic autoimmune disease clinically characterized by oral and ocular dryness, reflecting lymphocytic infiltration and subsequent exocrine gland dysfunction. However, during disease progression, any organ or mucosal surface may be involved. Thus, pSS presents as a heterogeneous non-organ-specific autoimmune entity, encompassing a wide spectrum of clinical manifestations, serological abnormalities, and scattered complications [1]. The incidence rate of pSS is estimated at about 6.92 per 100,000 person-years, with a female-to-male ratio in incidence data of 9:1 [2]. Prevalence rates vary widely between studies, but they are estimated at about 43.03 cases per 100,000 inhabitants when considering only population-based studies [2]. The peak incidence of pSS is in women aged 55–65 years [2].

Because pSS is a systemic autoimmune disorder, several autoantibodies are detected in pSS patients [3], of which antinuclear antibodies (ANA) are the most frequently detected (in up to 80% of patients); however, the most relevant autoantibodies are directed against Ro/SSA or La/SSB antigens [3]. According to the 2002 American-European Consensus Group (AECG) classification criteria [4], the diagnosis is based on the report of dry mouth and dry eyes by patients, salivary gland biopsy, sialography and/or scintigraphy, positive serology, and ophthalmic testing to confirm tear deficiency. The 2002 AECG criteria have specificity of 95.2% and sensitivity of 89.5% [4].

In 2012 and 2016, two sets of diagnostic criteria were proposed [5, 6]. However, salivary gland ultrasonography (SGUS) was not included in any set of diagnostic criteria even though some studies have indicated that it could replace scintigraphy, sialography and other imaging techniques in the diagnosis of pSS [7–11]. SGUS is simple, noninvasive, widely available, non-irradiating and less expensive than other imaging techniques [12]. Its use allows us to classify the echogenicity, homogeneity, degeneration, fibrosis and calcification of the glandular parenchyma.

In 1992, De Vita et al. [13] comparatively investigated SGUS abnormalities in patients with either primary or secondary Sjögren’s syndrome and controls. Scores of 0–3 were assigned to each pair of glands according to inhomogeneity, hypoechogenicity, size, and posterior borders of the parotid and submandibular glands, and this scoring system showed a sensitivity of 88.8% and a specificity of 84.6% for pSS [13]. Since then, there have been an increasing number of studies evaluating ultrasonography in the diagnosis of pSS. Hocevar et al. [14] evaluated new parameters in glandular tissue and suggested scores of 0–48 according to inhomogeneity, echogenicity, number of hypoechoic areas, and gland borders, achieving a sensitivity of 58.8% and a specificity of 98.7%. In a prospective cohort of patients with pSS, Cornec et al. [12] examined submandibular and parotid glands bilaterally and the findings were graded on a scale of 0–4, where grade 0 = normal, grade 1 = small hypoechogenic areas without echogenic bands, grade 2 = multiple hypoechogenic areas measuring < 2 mm with echogenic bands, grade 3 = multiple hypoechogenic areas measuring 2–6 mm with hyperechogenic bands, and grade 4 = multiple hypoechogenic areas measuring > 6 mm or multiple calcifications with echogenic bands. This scoring system achieved 62.8% sensitivity and 95.0% specificity [12]. In addition, a large number of studies have produced convincing data supporting the usefulness of SGUS for the prognostic stratification of patients with pSS, showing that patients with pathologic SGUS have positive serology, higher disease activity, and significantly more often several risk factors for lymphoma than patients with normal SGUS findings [15, 16].

New indices have been developed to objectively assess systemic and symptomatic manifestations in patients with pSS. The European League Against Rheumatism (EULAR) Sjögren’s syndrome disease activity index (ESSDAI) was developed in 2009 by consensus of a large group of worldwide experts from European and North American countries [17]. The ESSDAI includes 12 organ-specific domains (cutaneous, pulmonary, renal, articular, muscular, peripheral nervous system, central nervous system, hematological, glandular, constitutional, biological, and lymphadenopathy), and scores < 5 indicate low activity, scores ≥5 and ≤13 indicate moderate activity, and scores ≥14 indicate high activity [18].

The objective of the present study was to determine whether the severity of salivary gland involvement as assessed morphologically by SGUS or functionally by salivary flow could predict higher ESSDAI in patients with pSS.

## Materials and methods

### Participants

Seventy patients with pSS were selected from a cohort of 84 patients followed in the Departments of Internal Medicine and Ophthalmology at Universidade Federal de São Paulo from November 2013 to May 2016. The 2002 AECG criteria were used to select patients for inclusion. Exclusion criteria were hepatitis B or C, sarcoidosis, other connective tissue diseases, and current biological therapy. The following data were collected from each participant: previous medical history; complaints related to oral and ocular dryness; eye examination (ocular staining score and Schirmer’s test); serological tests including rheumatoid factor (nephelometry), ANA (indirect immunofluorescence), anti-Ro/SSA and anti-La/SSB (radial double immunodiffusion), and immunoglobulin G (IgG) levels (nephelometry); stimulated salivary flow rate; erythrocyte indices; urinalysis; and bilateral SGUS of the parotid and submandibular glands.

All included patients fulfilled the AECG classification criteria [4] and gave written informed consent to participate. The study was approved by the local ethics committee at Universidade Federal de São Paulo (CAAE 17092013.8.0000.5505).

### Ultrasonographic examination

SGUS was performed by the same investigator, an experienced radiologist, using a real-time scanner (Logiq P6; GE Healthcare, Waukesha, WI, USA) with a 12-MHz linear array transducer. Both the parotid and submandibular glands were scanned and echostructure of each gland was graded on a scale of 1 to 4. According to this scale, grade 1 = small hypoechogenic areas without echogenic bands, grade 2 = multiple hypoechogenic areas measuring < 2 mm with echogenic bands, grade 3 = multiple hypoechogenic areas measuring 2–6 mm with hyperechogenic bands, and grade 4 = multiple hypoechogenic areas measuring > 6 mm or multiple calcifications with echogenic bands [12]. Each patient received one score per gland and the highest score obtained was considered in the analysis.

### Salivary flow

The participants were asked to spit saliva into a graduated test tube for 15 minutes after salivation was stimulated with 2% citric acid applied to the lateral borders of the tongue. Stimulation with 2% citric acid followed the protocol described by Navazesh et al. [19]. This procedure was performed at the same time in the morning at normal room temperature and humidity and participants were asked not to eat/drink/smoke for at least 2 hours beforehand. The entire saliva sample was stored at −20°C and salivary and serum beta-2 microglobulin (β2M) levels were measured using an Abcam β2M ELISA kit.

### ESSDAI determination

The date of disease diagnosis was defined as the date of fulfillment of the 2002 AECG criteria as confirmed by the physician in charge of the patients in the cohort. Systemic involvement was defined according to the ESSDAI, which evaluates the following 12 domains or organ systems: constitutional, cutaneous, articular, muscular, lymphadenopathy, glandular, pulmonary, central nervous system, peripheral nervous system, hematological, renal, and biological [17].

### Statistical analysis

For analysis purposes, participants were divided into two groups according to SGUS score: score of 1 or 2 and score of 3 or 4. Descriptive statistics (mean, SD and 95% confidence interval [95%CI] for continuous variables and frequency and percentage for categorical variables) were used to characterize the patients in the two groups. Continuous variables were compared using Student’s *t* test (for variables with normal distribution) or the Mann-Whitney test (for variables with skewed distribution) and categorical variables were assessed using the chi-square test. Data analysis was performed using SPSS, version 19.0, and the level of statistical significance was set at 5%.

## Results

The sample consisted of 70 patients, 68 (97%) women and 2 (3%) men, with a mean (SD) age of 55.74 (11.89) years and median disease duration of 6.0 years (range, 2.0–10.0 years). The most common symptom was dryness of the mouth and/or eyes (100%).

The main serological features at diagnosis were ANA ≥1/80 in 58/70 (83%) patients, anti-Ro/SSA in 39/70 (58%), anti-La/SSB in 22/70 (31%), and rheumatoid factor in 38/60 (54%). The ESSDAI was 0–4 in 51/70 (72.9%), 5–14 in 18/70 (26%), and ≥15 in 1/70 (1%). An ESSDAI < 5 was found in 51 patients (72.9%), while an ESSDAI ≥5 was found in 19 patients (27.1%). Table 1 shows the characteristics of the study sample divided into two groups according to SGUS findings.

**Table 1:** Cohort characteristics.

Variables	Groups		
	Score 1 or 2 (n = 23)	Score 3 or 4 (n = 47)	p-value
Age (years), mean (SD)	57.3 (10.9)	55.0 (12.4)	0.434*
Sex M:F, n (%)	1 (4.3): 22 (95.7)	1 (2.1): 46 (97.9)	0.600**
Disease duration (years), mean (95%CI)	9.3 (6.5–12.2)	6.9 (5.2–8.5)	0.101*
ESSDAI, mean (95%CI)	1.65 (0.81–2.49)	3.89 (2.77–5.01)	0.013***
Categorical ESSDAI, n (%)			0.064**
ESSDAI < 5	20 (87.0)	31 (66.0)	
ESSDAI ≥ 5	3 (13.0)	16 (34.0)	
Salivary flow, mean (95%CI)	0.23 (0.14–0.32)	0.10 (0.07–0.13)	0.001***
ANA, n (%)			0.006**
Positive	15 (65.2)	43 (91.5)	
Negative	8 (34.8)	4 (8.5)	
Anti-Ro/SSA, n (%)			0.003**
Positive	7 (30.4)	32 (68.1)	
Negative	16 (69.6)	15 (31.9)	
Anti-La/SSB, n (%)			0.077**
Positive	4 (17.4)	18 (38.3)	
Negative	19 (82.6)	29 (61.7)	
RF, n (%)			0.034**
Positive	8 (34.8)	29 (61.7)	
Negative	15 (65.2)	18 (38.3)	
IgG, mean (95%CI)	1268.9 (1083.8–1454.0)	1543.6 (1363.5–1723.6)	0.281*
Categorical IgG, n (%)			0.077**
≤ 1600 mg/dL	19 (82.6)	29 (61.7)	
> 1600 mg/dL	4 (17.4)	18 (38.3)	
Salivary β2M, mean (95%CI)	3.18 (2.00–4.37)	3.98 (2.19–5.77)	0.547***
Serum β2M, mean (95%CI)	2.27 (0.95–3.59)	5.73 (2.56–8.89)	0.088***

ANA, antinuclear antibodies; β2M, beta-2 microglobulin; ESSDAI, European League Against Rheumatism (EULAR) Sjögren’s syndrome disease activity index; IgG, immunoglobulin G; RF, rheumatoid factor.

* Student’s *t* test

** Chi-square test

*** Mann-Whitney test

In order to relate SGUS findings to clinical and laboratory variables, patients were divided into two groups: SGUS score of 1 or 2 (n = 23, 33%) and SGUS score of 3 or 4 (n = 47, 67%). The group with scores 3 or 4 had proportionally more patients with an ESSDAI ≥5 (p = 0.064), with positive ANA (p = 0.006), and with positive rheumatoid factor (p = 0.034) than the group with scores 1 or 2. There was also an association between patients with SGUS scores of 3 or 4 and presence of anti-Ro/SSA (p = 0.003), but not of anti-La/SSB (Table 1).

Disease duration and age did not relate to the ESSDAI or SGUS score. There was no significant difference in the proportion of IgG > 1600 mg/dL between the two groups. However, a tendency to higher IgG levels was observed in the group with scores 3 or 4 than in the group with scores 1 or 2 (p<0.100). IgG levels did not relate to the ESSDAI. Patients with positive anti-Ro/SSA had proportionally more IgG > 1600 mg/dL (p = 0.014), but the same was not true for anti-La/SSB.

The relationship between salivary flow and SGUS score was significant, with patients with scores of 1 or 2 showing significantly higher salivary flow than patients with scores of 3 or 4 (p = 0.001). Salivary β2M levels did not relate to any variable (Table 1).

## Discussion

In the present study, we evaluated the prognostic value of SGUS in a cohort of 70 patients with pSS. These patients were selected from a cohort of 84 patients, and one criterion for exclusion was biologic or immunosuppressive therapy in the past 3 months. Most patients used hydroxychloroquine, omega 3, eye drops, and artificial saliva. We found an association of more severe SGUS scores (3 and 4) with the presence of anti-Ro/SSA antibodies, IgG levels > 1600 mg/dL, and an ESSDAI ≥5, which is consistent with the findings in the literature [15, 16]. There was no association with longer disease duration although a positive relationship was found between disease severity and low salivary flow. Although the new 2016 criteria withdrew the anti-La/SSB antibody from use due to its low specificity, in the present study, we observed that the anti-Ro/SSA group had higher SGUS scores than the anti-La/SSB group.

Since the study published in 1992 by De Vita et al. [13], SGUS has emerged as a promising tool for the diagnosis and prognostic stratification of patients with primary or secondary Sjögren’s syndrome. Some studies have reported that, although SGUS would be highly specific for the diagnosis of the disease, this is not sufficient to make it part of the diagnostic criteria [20]. However, SGUS is a feasible and inexpensive method that has proven useful for assessing major salivary gland involvement in Sjögren’s syndrome, exhibiting good diagnostic properties [8, 10, 12, 21]. The present findings confirm what has been reported in the literature.

In 2013, Cornec et al. [12] performed SGUS in a prospective cohort of patients with suspected pSS. SGUS was able to distinguish patients with pSS (n = 78) from controls (n = 80) with 62.8% sensitivity, 95.0% specificity, 92.5% positive predictive value, and 72.4% negative predictive value. We used the Cornec classification in the present study, which establishes four scores according to inhomogeneity and size of the anechoic area.

The relationship between SGUS scores and disease severity has been demonstrated in recent studies [11, 15, 16]. Hammenfors et al. [16] investigating 97 patients showed that those with severe changes determined by SGUS tended to be younger and had a higher degree of subjective and objective findings, indicating a patient subgroup with more severe pSS. The authors also found that increased dry mouth, fatigue and serological alterations were associated with more severe parenchymal findings on SGUS.

Niemelä et al. [11] found a strong correlation between the concentrations of ANA and anti-Ro/SSA and/or anti-La/SSB antibodies in 97% of patients with parenchymal heterogeneity of the salivary glands visible on SGUS. Theander and Mandl [15] recruited 105 patients with pSS and, using a simplified SGUS scoring system, showed that patients with a more severe SGUS score had a higher frequency of autoantibodies (anti-Ro/SSA and anti-La/SSB, ANA, and rheumatoid factor) and significantly higher levels of IgG than patients with normal SGUS findings. More interestingly, they found that disease activity, as measured by the ESSDAI, was significantly higher in these patients and there was a significant association between SGUS scores and lymphoma risk factors, including germinal center-like structures in the minor salivary gland, CD4+ T cell lymphopenia, reduced number of memory B cells in the circulation, monoclonal immunoglobulin in serum, presence of salivary gland swelling, purpura, and skin vasculitis.

SGUS may thus serve as a useful tool in selecting subgroups of patients who require closer follow-up. However, whether the time elapsed between the onset of symptoms and diagnosis is an important factor remains to be clarified. Future studies should therefore investigate serum biomarkers [22] in combination with noninvasive methods, such as SGUS, about their usefulness in the early detection of patients with pSS.

ESSDAI for pSS is a tool that helps clinicians evaluate disease activity [17]. The ESSDAI grades 12 organ-specific domains on a scale of severity of involvement, ranging from 0 (no activity) to 3 (high activity). These scores are then multiplied by an assigned weight factor, ranging from 1 to 6. Total scores range from 0 to 123. Most patients in studies using the ESSDAI as an outcome have shown a mean total score of 13 [23]. In our cohort, a mean (SD) ESSDAI of 3.15 (3.47) was observed, indicating a group with mild disease.

De Vita et al. [24], in a study on belimumab for pSS, reported a mean (SD) ESSDAI of 8.8 (7.4) at baseline. In the study by Kedor et al. [25], the mean (SD) ESSDAI at baseline was 5.5 (3.3). In a validation study conducted in Argentina, a mean ESSDAI of 5 was observed (ranging from 3 to 9), indicating the prevalence of patients with low disease activity or inactivity [26]. Studies conducted in different countries where the ESSDAI is applied have shown mean values of 4 (0 to 43) in an English cohort, 5.7 (0 to 29) in a French cohort, 3.18 (0 to 29) in a Dutch cohort, and 11.1 (0 to 37) in a Finnish cohort [23, 26–29]. In all these samples, the values indicate patients with low disease activity. In a study involving 62 patients with pSS for the transcultural adaptation of the ESSDAI into Brazilian Portuguese, the authors found a mean (SD) ESSDAI of 4.95 (6.73) [30]. In the present study, the ESSDAI was able to distinguish two groups of patients: 23 patients with a mean ESSDAI of 1.65 (95%CI 0.81–2.49) and 47 patients with a mean ESSDAI of 3.89 (95%CI 2.77–5.01).

Studies have reported that high serum β2M levels reflect clinical activity, systemic activity, and high lymphoma risk [29]. Salivary β2M levels, in turn, have been related to pSS in some studies as a biomarker for diagnostic purposes [31–33]. Thus, measurement of β2M levels in inflammatory fluids may provide a simple method for quantifying local activity in autoimmune states [34]. Elevated β2M concentrations were found in the saliva of patients with pSS and in the synovial fluid of patients with rheumatoid arthritis [34]. In addition, a correlation was found between elevated salivary β2M concentration and the degree of inflammation seen in the labial salivary gland biopsy [35].

In the present study, β2M levels were measured in serum and saliva in order to evaluate whether this biomarker would be able to detect disease severity in accordance with a high activity index, low salivary flow, and high SGUS score. However, our results were not consistent with a high ESSDAI, high SGUS score, or low salivary flow. A possible explanation for this may be the small sample size and absence of gland inflammatory reaction due to long disease duration. It is therefore a tempting prospect to test β2M levels in the early stages of the disease when there are no inflammatory changes in glandular tissue function.

Because our patients had a very low salivary secretion, stimulated saliva collection was required to obtain optimal amounts of saliva samples for β2M measurements. Some studies have demonstrated the reliability of stimulated salivary flow, providing a good application method [36]. Stimulated whole salivary flow correlated better with minor salivary gland pathology than with unstimulated salivary flow and may therefore serve as a noninvasive surrogate biomarker of inflammation and fibrosis as well as a measure of response to treatment in patients with pSS [37]. In the present study, citric acid was used on both sides of the tongue, three drops on each side, and collection began after the citric acid had been swallowed. Nevertheless, a better standardization of the collection and processing of saliva as well as further validation, refinement, and technical improvements will be required in studies aiming to identify salivary biomarkers [37].

Polyclonal hypergammaglobulinemia is one of the most characteristic laboratory abnormalities found in pSS. It reflects the polyclonal B-cell activation implicated in the pathogenesis of the disease and provides useful analytical data that may strengthen the diagnosis of pSS in patients with sicca syndrome [38]. Hypergammaglobulinemia is closely associated with the key immunological markers of Sjögren’s syndrome (rheumatoid factor, anti-Ro/SSA, and anti-La/SSB). Several studies have reported that up to 10% of patients with pSS may have associated monoclonal gammopathy of undetermined significance (MGUS). This gammopathy was included as a key marker of disease activity in the proposed ESSDAI [17]. In our cohort, 31.5% of patients with a more severe SGUS score had IgG levels > 1600 mg/dL.

This study has some limitations that should be taken into account when interpreting the results. The SGUS was performed by a single examiner who, despite having sufficient expertise in the examination, was not blinded to the diagnosis. However, the examiner was asked to assign only the score of severity of salivary gland involvement, according to the Cornec classification, and was blinded to all other disease or patient data. A labial salivary gland biopsy could have added more data to the evaluation of prognosis. Only 20% of our patients had labial salivary gland biopsies. In our department, a biopsy is performed only if the 2002 AECG criteria are not fulfilled.

In summary, our sample consisted of patients with pSS already diagnosed in the community hospital setting who were referred to our institution through different specialties related to the disease (dentists, ophthalmologists, rheumatologists, and otolaryngologists). This fact encourages us to believe that our results reflect a real-world scenario. In addition, some associations found in the present study could improve patient follow-up, such as patients with high SGUS scores need to be followed more closely as they are at increased risk of poor prognosis. It is also important to promote the application of the ESSDAI in clinical situations, since this index could measure the likelihood of poor outcomes more accurately.

Finally, it is recommended that further research be conducted in a large population, using SGUS as a tool to measure disease severity, for a prolonged observation time to determine other aspects of systemic manifestations that match additional characteristics of severity measured with the ESSDAI. We believe that SGUS is an affordable, convenient and reliable option to detect Sjögren’s syndrome, with the advantage of avoiding unnecessary radiation exposure as in sialography and scintigraphy.

## Conclusion

The associations observed in the present study between SGUS, severity of dryness symptoms, exocrine gland function, systemic autoantibodies, and disease activity indicate the usefulness of SGUS as a tool to assess salivary gland involvement in pSS. Further studies are needed to elucidate the potential role of major salivary gland ultrasound imaging as a diagnostic and prognostic tool in pSS.
